# Neurovascular Interaction and the Pathophysiology of Diabetic Retinopathy

**DOI:** 10.1155/2011/693426

**Published:** 2011-02-21

**Authors:** Haohua Qian, Harris Ripps

**Affiliations:** ^1^Department of Ophthalmology and Visual Sciences, University of Illinois College of Medicine, 1855 West Taylor Street, Chicago, IL 60612, USA; ^2^Visual Function Core, National Eye Institute, 49 Convent Drive, Room 2B04, MSC 4403, Bethesda, MD 20892, USA; ^3^Department of Physiology and Biophysics, University of Illinois College of Medicine, 1855 West Taylor Street, Chicago, IL 60612, USA; ^4^Department of Anatomy and Cell Biology, University of Illinois College of Medicine, 1855 West Taylor Street, Chicago, IL 60612, USA; ^5^Molecular Physiology Program, Marine Biological Laboratory, Woods Hole, MA 02543, USA

## Abstract

Diabetic retinopathy (DR) is the most severe of the several ocular complications of diabetes, and in the United States it is the leading cause of blindness among adults 20 to 74 years of age. Despite recent advances in our understanding of the pathogenesis of DR, there is a pressing need to develop novel therapeutic treatments that are both safe and efficacious. In the present paper, we identify a key mechanism involved in the development of the disease, namely, the interaction between neuronal and vascular activities. Numerous pathological conditions in the CNS have been linked to abnormalities in the relationship between these systems. We suggest that a similar situation arises in the diabetic retina, and we propose a logical strategy aimed at therapeutic intervention.

## 1. Introduction

Early Events in the Development of DR the ocular manifestations of diabetes can often strike with little warning, and despite efforts to prevent its occurrence, approximately 20,000 Americans go blind from diabetic retinopathy every year [1]. As with many diabetes-related conditions, high blood glucose levels (hyperglycemia) present as the main cause of diabetic retinopathy [2]. At an early stage, classified clinically as nonproliferative DR, there is a thickening of the capillary basement membranes and a loss of pericytes in the ocular vasculature [3]. Changes in the mechanical properties and permeability of the retinal blood vessels lead to the formation of microaneurysms (i.e., small outpouchings from the vessel wall) in capillaries within the inner nuclear layer [4]. Further vascular deterioration results in retinal hemorrhages, microretinal infarcts in the nerve fiber layer of the retina, deposits of cotton-wool spots, and abnormalities in the electrical activity of the retina [3, 5–7]. The progressive closure of retinal vessels produces localized areas of tissue ischemia, venous beading, and other intraretinal microvascular abnormalities that increase retinal hemorrhage and exudation. The advancement to proliferative diabetic retinopathy is viewed to be a consequence of tissue ischemia and subsequent upregulation of angiogenic growth factors, for example, vascular endothelial growth factor (VEGF), and vascular invasion of the inner retina. Clearly, the challenge is to find a therapeutic approach that can limit or perhaps prevent the onset of these serious complications. Here we present a rationale for a novel therapeutic approach based on the interaction between the retinal nerve cells and their blood supply. We suggest that the link between the blood supply of the inner retina and the concomitant changes in neural activity provides a mechanism that could be modified pharmacologically to prevent the onset of diabetes-induced retinopathy.

## 2. Factors Considered to Be Involved in the Development of DR 

A number of interrelated hyperglycemia-affected pathways have been identified in the pathobiology of diabetic complications, and a number of agents have been developed to interrupt pathways implicated in the pathogenesis of DR; (see [8] for review). Among the most actively studied are (1) oxidative stress [9–11], (2) polyol pathway activity [12, 13], (3) advanced glycation end-product (AGE) formation [14, 15], (4) activation of protein kinase C (PKC) isoforms [16, 17], and (5) increased hexosamine pathway flux [18]. However, the linkage between any particular pathway and the development of DR is still largely speculative. Although several promising drugs have been tested extensively, few have proven beneficial owing perhaps to the fact that treatment is typically implemented when there is already evidence of severe retinopathy. 

A new approach that could provide intervention earlier in the disease to delay or prevent the onset of retinopathy would clearly be of great benefit in the management of DR. We suggest that reestablishing the balance between neuronal activity and vascular function will suppress hypoxia in diabetic retinas and provide therapeutically beneficial effects in cases of DR. In this connection, it is important to recognize the link between the neural and vascular systems and their functional interdependence. This is clearly evident in the inner layers of the retina where the nerve cells are susceptible to the metabolic or hypoxic/ischemic vascular insult resulting from diabetes. In the sections that follow, we will present evidence of neurovascular coupling in the nervous system, describe the ways in which the regulation of neuronal activity could serve as a means of reducing hypoxic stress in the diabetic retina, and develop a rationale for a novel therapeutic approach based on the interaction between the retinal nerve cells and their blood supply. Specifically, we propose the use of a GABA_C_ receptor agonist, 5-methyl-I4AA, to suppress the neural activity of the inner retina, thereby establishing a normal neurovascular relationship to relieve diabetes-induced ischemia. 

**Figure 1:**
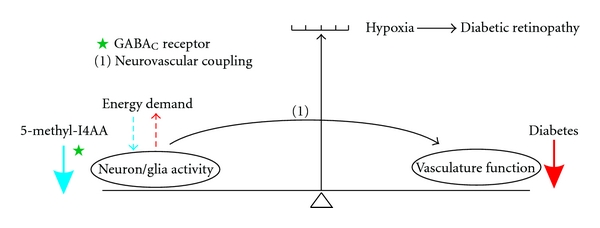
Schematic diagram illustrating (i) the normal balance between neuronal activity and vascular function and (ii) the action of 5-methyl-I4AA in preventing the development of diabetic retinopathy. Arrows at the right and left of the figure represent the opposing forces that upset (red arrow) or restore (blue arrow) the normal balance between vascular and neuronal function in diabetes. Diabetes disrupts the functional integrity of the vascular system of the inner retina (red arrow) and tilts the balance indicator toward the production of tissue hypoxia/ischemia that induces DR. The resultant imbalance in neurovascular coupling (pathway (1)) increases the energy demands of retinal neurons and glial cells to further exacerbate the condition (dashed red line). 5-methyl-I4AA, by activating the GABA_C_ receptors, suppresses the activity of inner retinal neurons (blue arrow) and reduces their metabolic demand (dashed blue line). This process reestablishes the normal balance of the neuron-vascular relationship. See text for additional details.

The essential features of this mechanism are illustrated schematically in Figure 1. A majority of the energy consumption in the nervous system is associated with neuronal activity and the cycling of neurotransmitters. Cell-cell and cell-matrix signals between neurons and glial cells trigger the regulatory mechanisms of the vascular system. Conversely, the vasculature supports neuronal activity and its metabolic demands. In normal circumstances, hemodynamic neurovascular coupling ensures the balance between neuronal activity and vascular function. Under diabetic conditions, however, the capacity of the vascular system is upset, thereby compromising the blood supply to the retina, causing tissue hypoxia, and creating an imbalance in neurovascular coupling. Prolonged tissue hypoxia leads eventually to the development of diabetic retinopathy. We propose that activating the GABA_C_ receptors on retinal neurons with 5-methyl-I4AA will suppress the activity of inner retinal neurons and reestablish a normal neurovascular relationship to relieve tissue hypoxia. Thus, treating the eye with 5-methyl-I4AA may be able to prevent or delay the development of retinopathy under diabetic condition.

## 3. NeuroVascular Interactions in the CNS

In most tissues, requirements for nutrients fluctuate in accordance with variations in activity. In the CNS, for example, there is extensive experimental evidence that cerebral blood flow is modulated by a variety of sensory stimuli. These findings, which have profoundly impacted our understanding of the relationship between increased neuronal activity and increased cerebral blood flow, have led to the mapping of brain activation now widely used in the diagnosis of brain pathologies [19]. Neurovascular coupling plays an important role in the maintenance of normal function in the nervous system, and defects in this relationship often lead to various forms of disease [20–22]. For example, stroke is now recognized as involving multiple cell types that disrupt the “neurovascular unit” [23, 24]. Ischemic damage results from the excessive activation of glutamate receptors, the recruitment of inflammatory cells, the overproduction of free radicals, and the initiation of apoptosis. It is now widely accepted that brain function involves the complex interactions between multiple cells types, including neurons, glial cells, and the microvascular endothelial cells comprising the cerebral vasculature [25, 26]. Thus, in addition to stroke, disruption of this neurovascular coupling has been implicated in Alzheimer's disease [27], epilepsy [28], Parkinson's disease, migraine, and other disorders of the CNS [20, 29]. Although a number of studies have focused on cellular and molecular mechanism of neurovascular coupling in the CNS, there are few that examine the relationship between neural activity and vascular function in the eyes of living animals (cf. [21, 30]).

## 4. Neurovascular Relationships in Mammalian Retina

The retina is arguably the most metabolically active tissue in the body, demanding the highest rate of blood supply per unit tissue weight [31]. However, the metabolism of its cellular components undergoes significant changes depending on both internal (e.g., blood pressure variations) and external (e.g., levels of photic exposure) factors. To meet these varying conditions, the retinal vasculature responds with regulatory mechanisms to control the blood supply to the tissue. Oxygen and nutrients are delivered to the retinal tissue by two separate pathways: the choroidal circulation supports cells of the outer retina (primarily photoreceptors and RPE), whereas the retinal circulation, subserved by the central retinal artery and vein, provides nourishment to inner retinal neurons (bipolar, amacrine, and ganglion cells) where the vascular abnormalities associated with diabetic retinopathy appear to be most pronounced. 

A number of studies have demonstrated that retinal blood circulation can be regulated in accordance with the activity of inner retinal neurons as well as in response to activation of glial cells [21]. In addition, using deoxyglucose as a tracer for metabolic activity, it has been shown that a flickering light stimulus selectively elevates the metabolic demand of cells in the inner retinal layers of rabbit [32] and monkey retinas [33]. Similarly, light-induced changes in retinal vascular oxygen tension have been recorded in rat eyes [34]. In addition, the increase in optic nerve head PO_2_ resulting from flickering light has been detected with phosphorescence imaging [35], and changes in intravascular PO_2_ have been reported based on the blood oxygen level-dependent signals obtained from magnetic resonance imaging [36]. These metabolic changes are associated with enhanced blood circulation in the retinal vasculature, which has been termed the visually-evoked hemodynamic response [37]. Thus, the activity of retinal neurons produces a metabolic demand that can lead to free-radical production in blood vessels compromised by DR.

## 5. Diabetic Effects on the Function of Retinal Neurons and Vasculature 

Diabetes has a significant impact on the functional status of the retinal vasculature; alterations in retinal blood flow have been observed in both animal models and human patients; (see [38] for review). Retinal oxygenation measured with fMRI techniques showed a reduction of retinal PO_2_ after the onset of hyperglycemia in rats made diabetic by ingestion of a high-galactose diet [39], and increased oxidative stress has been postulated as one of the major contributors in the development of diabetic retinopathy [40–42]. Diabetes and hyperglycemia increase the formation of reactive oxygen species (ROS), including nitric oxide (NO), superoxide (O_2_^. −^), and their product peroxynitrite (ONOO^−^) [43–46]. Increased formation of ROS is one of the early cell signals in diabetic retinopathy [46–48], and elevated nitrotyrosine levels in association with diabetes have been reported in a number of studies [46, 49, 50]. Diabetes impairs both neuronal activity and vascular function in the retina [51–54] such that vascular abnormalities cause an imbalance between the inner retinal blood supply and the metabolic demand of retinal neurons; these changes result in hypoxia. Conversely, a reduction in neural activity diminishes the stimulation of vascular responses. To what extent these functions are causally related is still an unanswered question [51], since the mechanisms that link neural impairment to the development of vascular abnormalities and retinal hypoxia in diabetes have not been adequately investigated. 

In human diabetic patients, there is a reduction in the hemodynamic response of retinal vessels to flicker light stimulation [39], which reflects most likely the underlying hyperglycemia-induced compromise of the microvasculature. In these circumstances, the endothelial cells of the retinal vasculature probably have an impaired ability to liberate endothelial nitric oxide synthase, which is important for their autoregulation [55]. Blood flow is also impaired by enhanced plasma viscosity, increased platelet aggregation, and decreased red blood cell deformability [56, 57]. These changes lead, in turn, to perfusion problems and local areas of retinal ischemia [58, 59], which have long been associated with the development of DR [60–62]. Changes in the blood supply to the inner retina exert a profound influence on the metabolism and activity of the retinal neurons, and consequently both the neuronal and vascular systems are compromised. However, whereas diabetic retinopathy is typically diagnosed clinically by abnormalities in the retinal microvasculature after prolonged hyperglycemia, there is well-documented evidence to indicate that neural deficits occur early in the course of the disease; (for reviews see; [52, 63]). For example, psychophysical tests have shown color vision defects and a reduction in contrast sensitivity [64], and neurosensory changes have been detected before the onset of observable retinopathy by means of the flash ERG [65] and multifocal ERG [66, 67]. In addition, degeneration of retinal ganglion cells has been reported in patients at early stages of diabetes [53], and our studies demonstrated diabetes-induced neural defects in 12-week old STZ-treated rats when measured by the ERG and with patch-clamp recordings from individual retinal neurons [68, 69]. Clearly, it is uncertain whether neural or vascular defects represent the earliest manifestations of the disease process.

## 6. The GABA Receptor and Oscillatory Potentials

The results of the studies cited above have led us to hypothesize that inhibiting the activity of inner retinal neurons will reduce the metabolic demands of the retinal cells and thereby diminish the diabetes-induced tissue hypoxia. Since hypoxic stress induces retinal cells to release a series of angiogenic factors that promote the clinical manifestations of DR, we sought an agent that would reduce neuronal activity in the inner retina, that is, slow their metabolic rate. Thus, it was necessary to identify an inhibitory neurotransmitter that suppressed the activity of inner retinal neurons, as well as an electrical response that originated from these neurons. Earlier studies have provided extensive information on these issues.

It is generally recognized that GABA (*γ*-aminobutyric acid) is the most prominent inhibitory neurotransmitter in the retina and CNS, whereas oscillatory potentials (OPs), a series of high-frequency wavelets superimposed on the ERG b-wave, are light-evoked electrical responses derived from inner retinal neurons. Numerous studies have shown that activation of GABA receptors diminishes the amplitude of OPs [70–72] and that OPs are very sensitive to disruption of GABAergic inhibitory neuronal pathways [70]. If, as we suggest, activation of GABA receptors will suppress neuronal activity, alleviate retinal hypoxia, and effectively reestablish the balance between the inner retinal blood supply and the metabolic demand of retinal neurons in DR, it then becomes essential that we identify the subtype of GABA receptor to be activated and the most effective agent to mediate the desired effect.

## 7. GABA and the Family of GABA Receptors

GABA is the main inhibitory neurotransmitter in both the CNS and retina [73–75]. Indeed, about 30–40% of all neuronal synapses within the mammalian brain are thought to be GABAergic [76–78]. In adult mammalian retina, where GABA mediates the majority of inhibitory synaptic transmission, amacrine cells are the main GABAergic inhibitory interneurons. These cells extend processes laterally to synapse with neighboring neurons in the inner plexiform layer, where they make inhibitory connections with bipolar cell terminals, ganglion cells, and other amacrine cells in the inner retina. In addition to other functional roles, GABAergic transmission is involved in the formation of complex receptive fields and directional sensitivity of ganglion cells [79–82].

The inhibitory effects of GABA are mediated by three major classes of GABA receptor present on postsynaptic neuronal membranes, termed GABA_A_, GABA_B_, and GABA_C_ receptors. Each has a distinct molecular structure and unique functional and pharmacological properties [83–87]. GABA_A_ receptors are a family of ligand-gated chloride channels that mediate rapid inhibitory reactions and have a diverse molecular composition consisting of mixtures of six subunits. However, they are not highly expressed on retinal bipolar cells, and, most importantly, they inactivate quickly. In contrast, GABA_B_ receptors belong to the G-protein-coupled receptor superfamily, whose inhibitory actions are mediated indirectly by second messengers that gate potassium and calcium channels. Although present on bipolar cells of lower vertebrates [88], GABA_B_ receptors are not seen in mammalian bipolar cells [89, 90]. 

Because of its localization and response properties, the GABA_C_ receptor is of special interest in the context of our hypothesis. It is composed for the most part of GABA *ρ* subunits, three of which (*ρ* 1–3) have been cloned from mammalian retinal cDNA libraries [91–93]. The GABA *ρ* subunits are distributed predominantly on retinal neurons, although their expression is also detected in other parts of the brain [85, 86, 94–96]. The unique physiological and pharmacological properties of the GABA_C_ receptors have been summarized in a series of reviews [75, 85–87, 97]. Particularly noteworthy is the fact that GABA_C_ receptors are located predominantly on the axon terminals of retinal bipolar cells where they contact inner retinal neurons [98, 99]. Equally significant is their slow kinetics of activation and deactivation in response to GABA, that is, the response is sustained with little sign of desensitization [90, 100, 101]. From a therapeutic standpoint, the restricted distribution reduces potential side effects, whereas its nondesensitizing properties allow for sustained inhibition of neuronal activity. Thus, the prolonged inhibitory effect on bipolar cell activity by externally applied agonists can be expected to significantly reduce the excitability of inner retinal neurons. Moreover, the GABA_C_ receptor is highly sensitive to agonists [100, 101], and, consequently, relatively low drug doses will be required to achieve the desired inhibition of inner retinal neurons.

## 8. Some Experimental Studies

Our proposal to use a GABA_C_ receptor agonist to activate this receptor subtype is not entirely conjectural. Several studies have shown that activation of GABA receptor-mediated inhibition can reduce hypoxia-induced glutamatergic activity to prevent cytotoxic effects on neurons in the CNS [102–104]. During the past decade, we have carried out an extensive series of studies on the localization, functional properties, and molecular composition of the GABA_C_ receptor [85, 101, 105–110] and we have examined the pharmacological properties of the GABA_C_ receptors on neurons in the retinas of normal and streptozotocin-induced diabetic rodents [68, 69]. As a first step in analyzing the effects of neuronal inhibition on retinal vasculature, we conducted a study to determine the efficacy of the GABA receptor agonist 5-methyl-I4AA on the response properties of retinal neurons and its effects both on visual performance and the retinal vasculature. It had been shown that methyl substitution at the 5 positions of the imidazole-4-acetic acid (I4AA) molecule acts as a specific, highly potent GABA_C_ receptor agonist [111]. A gift of 5-methyl-I4AA from Dr. Bente Frolund at University of Copenhagen, Denmark, allowed us to test its effects on rat retinal bipolar cells. 

**Figure 2:**
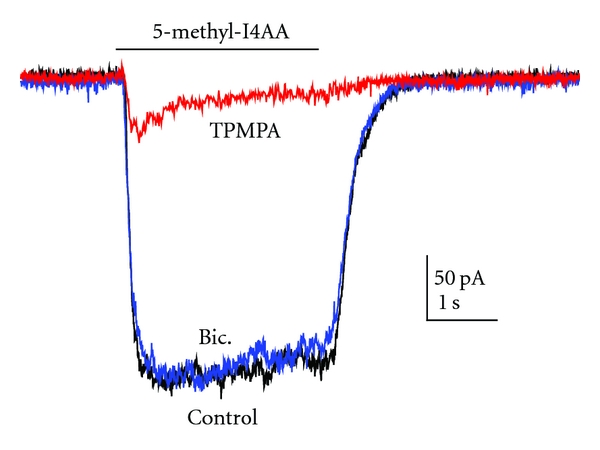
Membrane current response elicited from an isolated rat retinal bipolar cell by 5-methyl-I4AA (100 *μ*M) in normal saline (control), when coapplied with 100 *μ*M bicuculline, a GABA_A_ receptor antagonist (Bic.), or coapplied with 250 *μ*M TPMPA, a GABA_C_ receptor antagonist (TPMPA). Note that bicuculline has a negligible effect on the 5-methyl-I4AA-induced current, whereas TPMPA suppresses the response almost completely. Both actions confirm that there are few or no GABA_A_ receptors involved in 5-methyl-I4AA response elicited from rat bipolar cells.

**Figure 3:**
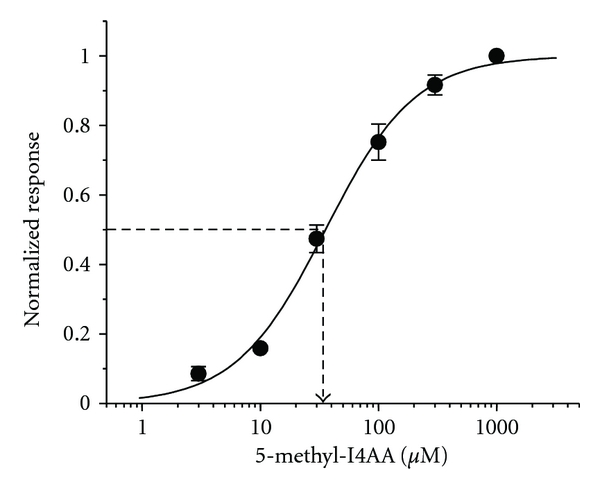
Dose-response relation of 5-methyl-I4AA-elicited current on rat retinal bipolar cells. Data were derived from the average of 6 cells and fit with a Hill equation with an EC_50_ of 35 *μ*M.

Figure 2 shows an example of current recordings obtained from isolated rat bipolar cells. With the membrane potential held at −60 mV, application of 100 *μ*M 5-methyl-I4AA induced a sustained inward current (Figure 2, Control). Although rat bipolar cells are said to contain both GABA_A_ and GABA_C_ receptors [68, 99, 112], the 5-methyl-I4AA-induced response was insensitive to the GABA_A_ receptor antagonist bicuculline, that is, the response to the coapplication of 100 *μ*M bicuculline with 5-methyl-I4AA was virtually the same as that elicited by 5-methyl-I4AA alone (Figure 2, Bic). In contrast, 5-methyl-I4AA responses were greatly reduced when coapplied with 250 *μ*M TPMPA, a GABA_C_ receptor antagonist (Figure 2, TPMPA). These findings indicate that the effect of 5-methyl-I4AA on rat bipolar cells is mediated almost exclusively by the GABA_C_-receptor. The dose-response relation for 5-methyl-I4AA induced current on rat bipolar cells is shown in Figure 3. The data were fit by a Hill equation with an EC_50_ of 35 *μ*M, a value that can serve as a guide for selecting drug dosages with which to test the effects of 5-methyl-I4AA in inhibiting neuronal activity. 

To determine the effect of diabetes on individual bipolar cells, we recorded GABA-elicited responses from isolated bipolar cells of diabetic rat retinas and compared them with the responses elicited from cells of age-matched normal retinas [68]. The results revealed that application of GABA generated a large, sustained inward current that deactivated slowly. When we coapplied bicuculline, a specific GABA_A_ receptor blocker, the response was reduced by about 20%, indicating that the response elicited from the bipolar cell is generated primarily by activation of GABA_C_ receptors, with only a small contribution from GABA_A_ receptors. Moreover, the dose-response relation of the GABA_C_ receptor-mediated response showed that the maximum current response was greater in cells derived from diabetic animals than those from age-matched controls and that the diabetic cells exhibited a higher GABA sensitivity than those from the normal control group. The enhanced GABA_C_ receptor activity in diabetic retinas indicates that activation of this receptor will maintain their inhibitory action in the retina even under hyperglycemic conditions. 

We also examined the distribution and expression of the GABA_C_ receptor subunits in diabetic retinas using immunohistochemical techniques [68]. We found, in agreement with earlier studies [98], that the majority of GABA_C_ receptors are expressed in the inner plexiform layer (IPL), most prominently on the bipolar cell terminals. The levels of GABA_C_ receptor expression in the diabetic retina were slightly higher than in control animals [69]. The fact that a similar GABA_C_ receptor expression pattern was observed in the diabetic retina indicated that long-term hyperglycemia does not alter the pattern of GABA_C_ receptor expression in the retina. Clearly, the GABA_C_ receptor could serve as a target for modulating neural activity under diabetic conditions.

**Figure 4:**
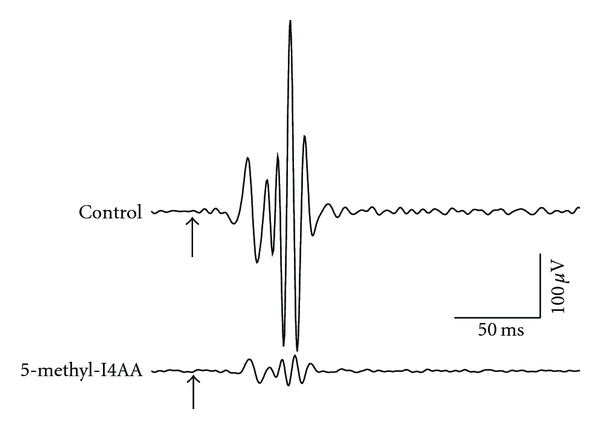
The effect of intravitreal injection of 5-methyl-I4AA (1 mM) on oscillatory potentials (OPs) recorded from rat eyes. The OPs, which arise from the activity of inner retinal neurons, were elicited after 1-hour dark adaptation; saline-injected eyes served as control. OPs were derived from digital band-pass filtered recordings (40–200 Hz) of the flash-evoked ERG. Arrows indicate the onset of a light flash (3 cd.s/m^2^).

In addition to extensive study of the localization, functional properties, and molecular structure of the GABA_C_ receptor, we examined a number of drug-induced responses in normal rats and those made diabetic by injection of streptozotocin. In order to demonstrate the efficacy of 5-methyl-I4AA on the activity of inner retinal neurons, we recorded the oscillatory potentials (OPs) of the electroretinogram (ERG) in response to a brief light flash (Figure 4). These components of the ERG, a series of wavelets that ride on the ascending phase of the ERG b-wave [113, 114], provide a noninvasive measure of the electrical response of neurons in the inner retina, where GABAergic mechanisms play a critical role in their generation [71]. Figure 4 compares the OPs elicited from a normal rat eye injected with saline (control) with those from the fellow eye that had received an intravitreal injection of 5-methyl-I4AA (1 mM calculated vitreous concentration). It is clear that OPs isolated from the complex ERG waveform using a digital zero-phase band-pass filter (40–200 Hz) were greatly reduced by intravitreal injection of 1 mM 5-methyl-I4AA (on average to about 23% of the response of saline-injected eyes).

Before suggesting the use of any drug, it is of paramount importance to demonstrate that it does no harm. We therefore performed an experiment to determine whether intravitreal injections of 5-methyl-I4AA (12 mM) into both eyes of two normal rats had any deleterious effect on the animals' visual performance. Using a virtual optomotor system (OptoMetry [115]) in Dr. Zhuo-hua Pan's laboratory (Wayne State University, Detroit, MI), we measured optomotor responses 24 hours after injection and compared the results with those obtained from two uninjected rats that served as control. Contrast sensitivity, measured at 6 spatial frequencies, was similar for the four animals, indicating that 5-methyl-I4AA did not grossly interfere with visual function (data not shown). It was not possible at this stage to determine whether more subtle changes in vision had occurred.

**Figure 5:**
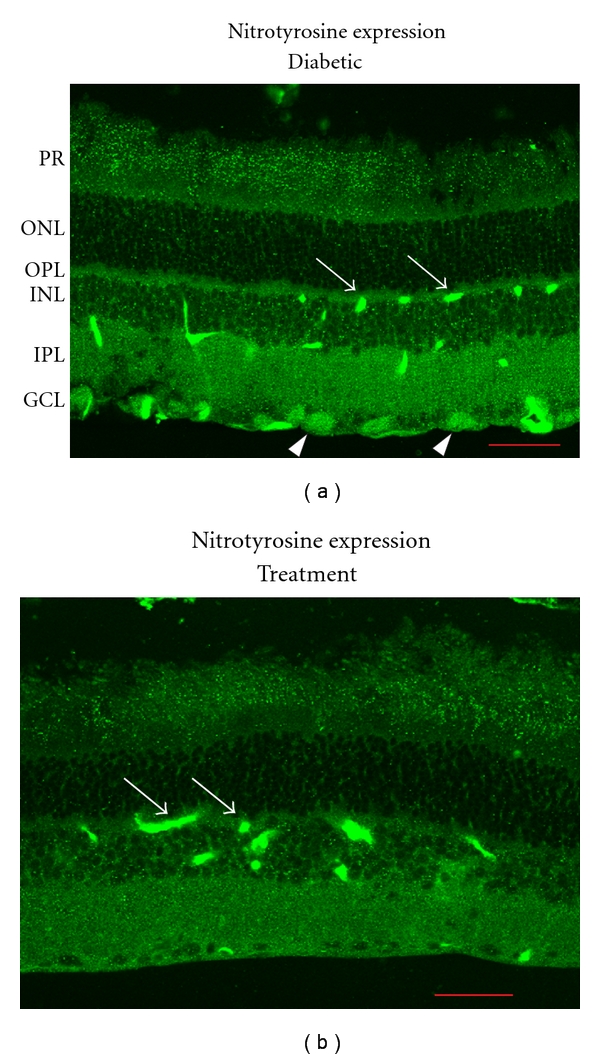
Nitrotyrosine expression in diabetic rat retina. Eyes were treated with saline (b) or with 5-methyl-I4AA (treatment). Nitrotyrosine expression was revealed by immunostaining with a polyclonal antibody (Chemicon) on cryosections of the retina. Arrows point to retinal blood vessels in both retinas. Arrowheads show staining of retinal ganglion cells in the diabetic retina but not in the treated retina. PR: photoreceptor layer, ONL: outer nuclear layer, OPL: outer plexiform layer, INL: inner nuclear layer, IPL: inner plexiform layer, and GCL: ganglion cell layer. Scale bar: 50 *μ*m.

**Figure 6:**
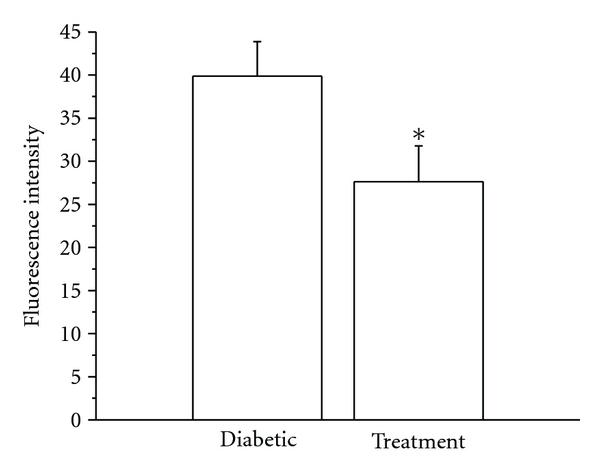
Bar graph illustrates nitrotyrosine fluorescence intensity in diabetic retina and those treated with 5-methyl-I4AA. Measurements from 3 animals show a significant reduction in fluorescence intensity (asterisk) after 5-methyl-I4AA.

We carried out a pilot experiment to examine the protective effects of intravitreal injection of 5-methyl-I4AA in 3 diabetic rats. 12 weeks after induction of diabetes with STZ, one eye of each animal was injected with 5-methyl-I4AA (vitreous concentration of about 1 mM), whereas the other eye was injected with saline and served as control. After 4 weeks of drug administration (one injection per week), rats were sacrificed and the retinas were isolated. The expression of diabetes-induced biomarkers was examined by immunohistochemistry and real-time RT-PCR techniques. As noted earlier, tyrosine nitration has been used as a biomarker for the development of diabetic retinopathy [46, 49, 50, 116], and thus changes in nitrotyrosine expression can be used to evaluate the protective role of GABA_C_ receptor agonists in the diabetic retina. As shown in Figure 5, we observed an increase in tyrosine nitration in the diabetic retina and found that application of 5-methyl-I4AA reduced its level of expression. In the diabetic animal (Figure 5(a)), the highest expression of nitrotyrosine was observed near retinal blood vessels (arrows) and in the cell bodies of retinal ganglion cells (arrowheads). Enhanced nitrotyrosine expression was also seen in the IPL although the fluorescence was less intense. After treatment with 5-methyl-I4AA (Figure 5(b)), an intense nitrotyrosine signal persisted in blood vessel areas (arrows), whereas the expression in retinal ganglion cell bodies and in IPL was significantly reduced. We used a published protocol to quantify the level of nitrotyrosine expression in the IPL [117], and four retinal sections from each of 3 diabetic rats were examined. Averaged results shown in Figure 6 confirmed that 5-methyl-I4AA treatment produced a significant reduction in nitrotyrosine expression (*P* < .05).

**Figure 7:**
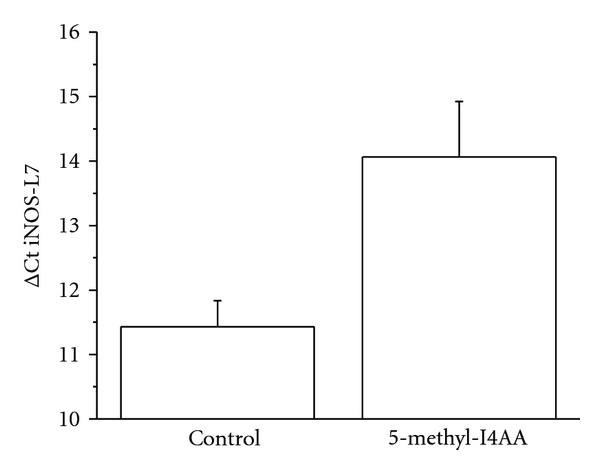
Real-time RT-PCR measurements of iNOS expression in the retinas of diabetic and 5-methyl-I4AA-treated rat eyes. The amount of RNA in each sample was determined by the value of Ct, the cycle number for the amount of PCR product needed to reach the fluorescence threshold. When normalized to L7 expression, a housekeeping gene of 60S ribosomal protein, the expression level of iNOS in diabetic retina was significantly reduced (i.e., a higher ΔCt value) after treatment with the GABA_C_ receptor agonist.

Lastly, we examined iNOS expression, another biomarker of the diabetic retina [116, 118–121]. Using real-time RT-PCR techniques, we measured iNOS expression in 3 diabetic rats receiving 5-methyl-I4AA treatment in one eye, whereas the fellow eye was injected with saline and served as control [69]. After 4 weeks of drug administration (one injection per week), the expression level of iNOS in diabetic retina was significantly reduced after treatment with the GABA_C_ receptor agonist, that is, application of 5-methyl-I4AA exhibited a protective effect on the diabetes-induced signal in rat retina (Figure 7). 

The findings obtained in our animal studies provide good evidence that a GABA_C_ agonist affords a form of pharmacological intervention that is capable of reestablishing the balance between the blood supply and neural activity and thus relieve hypoxia in the diabetic retina. The feasibility of this novel therapeutic approach in preventing or delaying the onset of retinopathy in diabetes should be further tested in animal models. If successful, the proposed therapy will have high translational value for the treatment of human patients. It is also noteworthy that reducing neural activity by activating GABA receptors in the retina may prove beneficial for preserving visual function under other pathological conditions, such as glaucoma. Clinically, glaucoma manifests as a loss of retinal ganglion cells and is thought to be caused in large part by glutamate-induced excitotoxicity. Activating the GABA_C_ receptors on bipolar cell terminals will be an effective means of reducing the release of glutamate, thus providing the ganglion cells with a measure of protection from exposure to cytotoxic levels of glutamate.

## 9. Some Issues for Consideration with This Pharmacological Approach

Although no adverse effects were seen with the introduction of 5-methyl-I4AA, there are reports that activation of GABA receptors could exacerbate the neuronal injury induced by excitatory agents or oxygen-glucose deprivation, especially for neurons at an early embryonic stage [122, 123]. This is probably related to the high intracellular Cl^−^ concentration of immature neurons, on which GABA often has an excitatory action. Indeed, it has been shown that GABA is protective for mature cortical neurons under hypoxia but toxic for immature cells [124]. Thus, the unique properties of the GABA_C_ receptor expressed in retinal neurons could serve as an ideal target for protecting against hypoxia associated with diabetes. 

GABA_C_ receptor agonists probably have a limited capacity for regulating the metabolic rate of retinal neurons, since a large component of the energy consumed by the cells provides for their “housekeeping” needs. However, it is unlikely that activating the GABA receptor on retinal neurons will shut down completely the metabolism of the cell. This could be the reason for a recent report citing the inability of a GABA receptor agonist in preventing ischemia-reperfusion injury in the retina [125]. On the other hand, diabetes is a chronic disease, and the hypoxia induced by diabetes in the retina is relatively mild. Reducing neuronal activity and metabolic demand in the diabetic retina could profoundly affect the development of DR.

These considerations aside, 5-methyl-I4AA is clearly a highly potent agent for activating the GABA_C_ receptor and should be potentially a useful drug for suppressing or delaying the onset of diabetic retinopathy. The novel approach for preventing the development of diabetic retinopathy that we propose, if more extensive studies in animal models confirm its beneficial effects, will have important and immediate translational value for treating human diabetic patients.
